# Green Synthesis of Gold Nanoparticles and Study of Their Inhibitory Effect on Bulk Cancer Cells and Cancer Stem Cells in Breast Carcinoma

**DOI:** 10.3390/nano12193324

**Published:** 2022-09-24

**Authors:** Jihui Wang, Na Liu, Qing Su, Yulong Lv, Chang Yang, Honglei Zhan

**Affiliations:** 1 School of Chemical Engineering and Energy Technology, Dongguan University of Technology, Dongguan 523808, China; 2 Department of Bioengineering, School of Bioengineering, Dalian Polytechnic University, Dalian 116034, China

**Keywords:** gold nanoparticles, hyaluronic acid, green synthesis, photothermal conversion, cancer stem cells, cancer therapy

## Abstract

Chemo-resistance from cancer stem cells (CSCs) subpopulation is a current issue in cancer treatment. It is important to select alternative therapies to efficiently eradicate both bulk cancer cells and CSCs. Here, gold nanoparticles (AuNPs) have been selected regarding their biocompatibility, facile and controllable synthesis, potent anti-cancer activity and photothermal conversion performance. We reported a green synthesis of functionalized AuNPs using hyaluronic acid (HA) as a reductant, capping, stabilizing and hydrophilic substance. The resultant AuNPs were spherical-shaped with an average diameter of around 30 nm. These AuNPs displayed improved physico-chemical (yield, stability, photothermal effect) and biological properties (cellular uptake, cytotoxicity and apoptotic effect) against bulk MDA-MB-231 cells, in comparison with other organic anti-cancer drugs. The intensified bioactivity was dependent on a mitochondria-mediated cascade, reflected by the damage in mitochondria, oxidative stress, intensified Caspase 3 activity and increased/decreased expression of certain pro-apoptotic (*Bax*, *P53*, *Caspase 3*)/anti-apoptotic (*Bcl-2*) genes. Moreover, these AuNPs posed a dramatically improved inhibitory effect in cell viability and self-renewable capacity on CSC subpopulation. All the results were attributed from the nano-scaled structure of AuNPs and combined effect from NIR-induced hyperthermia. In addition, the biocompatible nature of these AuNPs supported them to be a potential candidate in the development of novel chemotherapeutic drugs.

## 1. Introduction

The WHO has declared that cancer is still the most threatening disease causing mortality over the world, posing huge burden on human life, health, psychology and economy. In particular, breast carcinoma is the most frequently diagnosed malignant tumor for women [1,2,3]. Although chemotherapy is the most common treatment method, its therapeutic outcome is not satisfactory. The majority of patients undergo low therapeutic index, recurrence or metastasis, resulting from low bioavailability and great toxic/side effects from a couple of organic anti-cancer agents with multi-drug resistance, owing to the frequent single drug administration and existence of a small population of cancer stem cells (CSC) [4,5,6,7,8,9]. Extensive research proved that tumors are made up of both bulk differentiated cancer cells and undifferentiated CSCs. CSCs are able to self-renew and are responsible for cancer generation, development, relapse, metastasis and chemo-resistance [10,11]. In view of the challenges and difficulties above, it is important and necessary to select alternative therapies or novel chemotherapeutic agents to efficiently eradicate both bulk cancer cells and CSCs during cancer therapy [12,13].

Biomedical nanomaterials are currently being developed, with huge potential in the field of nanomedicine. Among them, noble metal (gold, silver or platinum) nanoparticles with inherent anti-cancer activity should be further explored as promising alternatives instead of current organic biopharmaceuticals on tumor treatment [14]. Gold nanoparticles (AuNPs) have been extensively preferred in the field of nanomedicine than bulk solid counterparts due to their unique physical and biological characteristics from great surface-to-volume ratio. To be specific, AuNPs exhibit a binding capacity with biomolecules, potent anti-angiogenic and anti-cancer activity, and their synthesis is facile and controllable [15,16,17,18]. As compared to other metal-based drugs, such as silver nanoparticles (AgNPs) and platinum-based drugs, AuNPs show least toxicity to animal and microorganism cells. Although AgNPs also demonstrate huge potential in cancer therapy due to their strong anti-cancer and anti-angiogenic properties, AgNPs and intracellular released Ag^+^ are potential toxic agents for normal cells. Moreover, AuNPs display superior photophysical property from their localized surface plasmon resonance (SPR) than other plasmonic noble metal NPs, that is, they have the greatest photothermal conversion efficiency upon exposure to NIR laser, thus combining chemotherapy and hyperthermia treatment to kill both bulk cancer cells and CSCs [19,20]. It is worth mentioning that AuNPs demonstrated their anti-cancer effect via various actions, such as DNA damage, interference in cell cycles, inhibition in thioredoxin reductase and proteasome and regulation in specific kinases. These multiple modes of action are vital for AuNPs to show improved anti-cancer activity, especially against platinum-resistant or multi-drug resistant cancer cells, because platinum-based drugs that exert their cytotoxicity mainly via DNA targeting and frequent single drug administration would induce the resistant mechanism of action from tumors. In consequence, AuNPs have been selected to handle cancer therapy and eliminate CSCs in this study.

AuNPs can be synthesized via reducing Au^3+^ to Au^0^ using chemical reducing agent, such as citrate or sodium borohydride, and stabilized by CTAB and so on. However, citrate-reduced AuNPs proved to exert toxicity against normal human endothelial cells, such as HDMEC and hCMEC [21]. On the other hand, the physical synthesis of AuNPs requires costly instrument and complicated operation procedures. To avoid these weaknesses, the green synthesis of AuNPs in biological ways, referring to utilization of living organisms, plan extract or other natural products, seems to be an effective method. This method could generate environmentally harmless AuNPs, showing low side/toxic effect during clinical application [22].

As was known that different reductants always synthesize AuNPs with different physico-chemical and biological properties. In this study, a biodegradable natural negatively charged polysaccharide, hyaluronic acid (HA), was used to synthesize AuNPs. The residual reductive groups in HA-reduced Au^3+^ to a zero-valent Au atom, and AuNPs were subsequently generated via Au atom aggregation. Meanwhile, HA could serve as a capping agent based on their hydroxyl groups, thus stabilizing newly produced AuNPs. Except for that, HA could specifically recognize and bind to CD44 receptors, which are overexpressed on surface of CSCs and bulk cancer cells. In a word, HA could be utilized as reductant, capping, stabilizing, hydrophilic and targeting agents [23,24,25,26,27], making AuNPs versatile.

We have successfully constructed HA-capped AuNPs when HA reacted with Au^3+^ under optimal conditions. These novel AuNPs showed good physical characteristics, biocompatibility and strong cytotoxic and apoptotic effect towards bulk cancer cells. In addition, they displayed remarkable inhibitory effect against the viability and self-renewable capacity of CSCs. The relevant mechanism of the action of these HA-capped AuNPs was also explored.

## 2. Materials and Methods

### 2.1. Cells, Materials and Chemicals

Gold (III) chloride trihydrate (HAuCl_4_·3H_2_O, 99%) and HA (MW 4000) powders were bought from Meilunbio company (Dalian, China); Hydrochloric acid and nitric acid was obtained from Kermel Chemical Reagent Co., Ltd. (Tianjin, China); MDA-MB-231 and normal L929 cells were all obtained from ATCC (Manassas, VA, USA); Cell culture and RT-PCR related chemicals were all purchased from TransGene Biotech Co., Ltd. (Beijing, China); MTT, MitoTracker Red CMXRos, DCFH-DA, DiI, Hoechst, AnnexinV-FITC/PI kit were all provided by Meilunbio Company (Dalian, China); Cationic fluorescent indicator (JC-1) and Caspase 3 assay kit were obtained from Dalian Saituo Biotechnology Co., Ltd. (Dalian, China); Western blot reagents and materials were all bought from Dalian Bogelin Biotechnology Co., Ltd. (Dalian, China); Monoclonal antibodies were purchased from Dalian Wanze Biotechnology Co., Ltd. (Dalian, China); PCR primers were synthesized by Sangon Biotech Corporation (Shanghai, China); B27, epidermal growth factor (EGF) and basic fibroblast growth factor (bFGF) were purchased from Millipore company (Burlington, MA, USA).

The human breast carcinoma MDA-MB-231 cells and normal mice fibroblasts L929 were maintained in DMEM medium, supplemented with 10% FBS and 1% penicillin/streptomycin solution at 37 °C in a cell incubator (95% humidity, 5% CO_2_). The isolation and cultivation of CSC subpopulation in MDA-MB-231 cells was according to a spheroid formation assay. Unlike bulk breast cancer cells, only fractions of CSCs are able to survive, proliferate and form mammospheres under certain (non-adherent, serum-free) conditions [28]. MDA-MB-231 cells with a density of 2 × 10^4^ cells/well were seeded into ultra-low attachment 24-well plate and cultivated in serum-free DMEM medium with 1×B27, 20 ng/mL EGF and 20 ng/mL bFGF at 37 °C in a CO_2_ incubator (95% humidity, 5% CO_2_). The cells were kept cultivation for 10 days and mammospheres were monitored and observed under an inverted light microscope. Those mammospheres with >50 μm diameters were counted as positive. The stemness nature of these mammospheres were identified by detecting gene expression levels of *Nanog* and *Sox-2*.

### 2.2. Green Synthesis of HA-Capped AuNPs and Their Characterization

HA-capped AuNPs were constructed, referring to previously reported method with proper modifications [29]. Aliquots (100, 150, 200 µL) of HAuCl_4_·3H_2_O solution (50 mM) were transferred dropwise to 1 mL HA solution (10 mg/mL) and final system volume was up to 10 mL by adding distilled water. The pH of these mixtures were adjusted to 9 with 1 mM Na_2_CO_3_ and then subjected to mild stirring for 3 h at 70 °C in dark. As a result, different batches of HA-capped AuNPs with different (1/2, 1/3, 1/4) HA/Au^3+^ molar ratios were obtained, indicated by the appearance of a ruby red/purple/dark red color. These resultant nanoparticles were centrifuged at 12,000 rpm for 1 h, washed 3–5 times with DI water and then re-suspended in known volume of DI water. 

Those different batches of HA-capped AuNPs were subjected to specific characterization, in order to certify the optimal synthesis conditions. To be specific, Au content in HA-capped AuNPs was quantified by an ICP-OES assay and relevant yield rate could be figured out. Their respective average particle sizes, PDI values and zeta potentials were determined based on DLS measurement (Nano-ZS90 Zetasizer, Malvern Instruments Corporation, UK). UV-vis spectra (400–800 nm) of different batches of AuNPs were measured to monitor the reduction of Au^3+^ to Au^0^ and validate the position and intensity of SPR band (Multiskan GO, Thermo Scientific Corporation, UK). SEM imaging (JEOL JSM-7800 F, Tokyo, Japan) and EDX analysis was performed for identifying the size, shape and elemental composition of these AuNPs. Chemical composition and interaction in HA-capped AuNPs and crude HA was determined by FTIR spectroscopy within a range of 450–2000 cm^−1^ (Nicolet Nexus 670, USA). To evaluate the aqueous dispersity and storage stability, AuNPs suspension (Au conc. = 75 µg/mL) was left and photographed at room temperature and 4 °C for 15 days, respectively. Aliquots of the suspension were sampled at pre-determined intervals to monitor the change in SPR band intensity. To examine the photothermal conversion efficiency, HA-capped AuNPs suspension in cell culture medium (200 µL) with different Au content (0–50 µg/mL) was exposed to an 808 nm laser (MDL-H-808-5W, Changchun, China) under various irradiation powers (1, 2 and 3 W/cm^2^) and durations (0–10 min). The control group (pure cell culture medium) were also subjected to the same NIR irradiation treatment. The ultimate equilibrium temperatures of each suspension were recorded by a digital thermometer. 

### 2.3. Level of Cellular Uptake of HA-Capped AuNPs in MDA-MB-231 Cells 

Human breast carcinoma MDA-MB-231 cells were cultured in 24-well plate with an initial seeding density of 1 × 10^4^ cells/well. Cell culture medium (DMEM) containing 20 µg/mL HA-capped AuNPs was added to the wells when reaching 80–90% confluence. Cells without AuNPs treatment were denoted as a negative group. At pre-determined time points (1, 3, 5, 7, 10, 24 h), cells in each group were washed with pre-cooled 1×PBS for 3–5 times and 1 mL of aqua regia (HCl/HNO_3_ at 3/1) was then added and incubated for 1 h, in order to dissolve and extract the intracellular Au. The mixture was diluted by DI water and the Au content in cell lysate was quantified by ICP-OES measurement. Cellular uptake rate of HA-capped AuNPs in each group was figured out through dividing extracted Au by initial added AuNPs [30,31]. In a typical experiment, MDA-MB-231 cells were first seeded onto a polylysine pre-treated glass coverslip at the bottom of the well. After incubation with HA-capped AuNPs (20 µg/mL) for 24 h, MDA-MB-231 cells were washed and fixed with 2.5% glutaraldehyde solution for 1 h at 4 °C. The fixed cells were washed again and gradually dehydrated with 30%, 50%, 70%, 80%, 90% and 100% ethanol for 5 min. Afterwards, the cells were treated with 100% ethanol for extra 10 min before observation by SEM technique.

### 2.4. Study of Cytotoxic Effect of HA-Capped AuNPs on MDA-MB-231 and L929 Cells

Human breast cancer cells MDA-MB-231 and mice normal L929 cells were seeded into 96-well cell culture plates at a concentration of 5 × 10^3^ cells/mL and cultivated for 24 h under optimal conditions. After discarding the cell culture medium, HA-capped AuNPs suspended in DMEM medium at different Au content (0–80 µg/mL) were added into each well. After 24 h incubation, cell viabilities from different groups were measured based on a classical MTT reduction experiment [30,31]. For comparison, a broad-spectrum chemotherapeutic drug Camptothecin (CPT) was administered under the same concertation range. CPT was first dissolved in DMSO and further diluted by DMEM before introduction to the cells. The final concentration of DMSO in the mixture did not exceed 0.1% (*v*/*v*), which is nontoxic to cells. To explore the combined effect of AuNPs and hyperthermia, in a separate group, after 20 h of AuNPs incubation, extra NIR irradiation (3 W/cm^2^ for 1 min) was performed. These NIR irradiated cells were further incubated with AuNPs suspension for 4 h. Cells without any drug/NIR treatment were regarded as a negative control. Cells only treated by NIR irradiation under the same condition were also studied to explore the direct impact of NIR laser on cells viability. The percentage of cell inhibition was determined and IC_50_ values were figured out.

### 2.5. Biocompatibility ASSESSMENt by a Hemolysis Test

The biocompatibility of HA-capped AuNPs was evaluated according to a classical hemolysis assay, as described previously [30,31]. Briefly, HA-capped AuNPs suspensions at different Au concentration (30, 50 µg/mL) and CPT suspension at 30 µg/mL were incubated with Kunming mice erythrocytes suspension (2 × 10^10^ cells/mL) for 1 h at 37°C, respectively. CPT was first dissolved in DMSO and further diluted by PBS before incubation. The resultant mixtures were centrifuged at 400 g for 10 min. OD values of the supernatant in each group was spectroscopically measured at 565 nm. For comparison, 1 × PBS and DI water-treated erythrocytes suspensions were regarded as a negative and a positive control, respectively. Relevant hemolytic rate was finally calculated. 

### 2.6. Apoptotic Activity of HA-Capped AuNPs and Underlying Mechanism

#### 2.6.1. Apoptosis and Necrosis

The apoptotic activity of HA-capped AuNPs with/without NIR laser treatment was examined using a classical AnnexinV-FITC/PI double staining method [29]. MDA-MB-231 cells were seeded into 24-well cell culture plates at a concentration of 1 × 10^4^ cells/well and cultivated overnight under optimal conditions. After removing the cell culture medium, HA-capped AuNPs DMEM suspensions with different Au content (5, 30 µg/mL) were added into each well and incubated for 24 h. For AuNPs+L group, after 20 h of AuNPs incubation, extra NIR irradiation (3 W/cm^2^, 1 min) was applied onto these cells. These NIR irradiated cells were further incubated with AuNPs suspension for 4 h. After that, cells were washed and stained by AnnexinV-FITC (5 μL) and PI (10 μL) solution for 15 min in dark. Necrotic and apoptotic cells were visualized under a fluorescence microscope (Olympus IX81, Tokyo, Japan). Cells lack of AuNPs or laser treatment were regarded as a negative control. 

#### 2.6.2. Nuclear and Membrane Damage

To capture the destructive activity on cell membrane and nucleus by HA-capped AuNPs, Hoechst 33,342 and DiI double staining was performed in MDA-MB-231 cells [29]. The procedures regarding cells seeding, cultivation and AuNPs/NIR treatment was the same as section Apoptosis and necrosis. After that, cells were washed and fixed by 4% formaldehyde solution for 1 h. The fixed cells were subjected to double-staining with Hoechst 33,342 (2 μg/mL, 100 μL) and DiI (5 μg/mL, 100 μL) solution for 0.5 h in darkness. These dye-labeled nucleus and membranes were visualized and compared under a fluorescence microscope (Olympus IX81, Tokyo, Japan). 

#### 2.6.3. Mitochondria Morphology and Mitochondrial Membrane Potential (MMP)

To explore the impact of AuNPs on mitochondria, MitoTracker Red CMXRos and JC-1 staining were performed to monitor the change in mitochondria morphology and MMP, respectively [32]. The procedures regarding cells seeding, cultivation and AuNPs/NIR treatment were the same as section Apoptosis and necrosis. After that, cells were washed and incubated with 100 nM MitoTracker Red CMXRos solution for 15 min under 37 °C. These dye-labelled mitochondria were visualized and compared under a fluorescence microscope. For MMP determination, after the same AuNPs/NIR treatment procedures, cells in each group were washed and incubated with 10 µM JC-1 working solution for 20 min under 37 °C. Fluorescent JC-1 aggregates and monomers were observed under a fluorescence microscope (Olympus IX81, Tokyo, Japan). MMP was defined as the rate of JC-1 aggregates to monomers. 

#### 2.6.4. ROS Activity 

To explore the activity of HA-capped AuNPs in ROS formation, a standard DCFH-DA assay was carried out, as previously reported [29]. The procedures regarding cells seeding, cultivation and AuNPs/NIR treatment was the same as section Apoptosis and necrosis. After that, cells were washed several times and incubated with 10 μM DCFH-DA solution for 20 min under 37 °C. Cells treated with 0.09% H_2_O_2_ solution were used as a positive group and those without AuNPs or NIR treatment were regarded as a negative group. The fluorescence intensity of these labelled cells were monitored and analyzed by a fluorescence microscope (Olympus IX81, Tokyo, Japan). 

#### 2.6.5. Caspase 3 Activity

The apoptotic activity of HA-capped AuNPs with/without NIR irradiation was further determined by Caspase 3 activity measurement, as described earlier [29]. The procedures regarding cells seeding, cultivation and AuNPs/NIR treatment was the same as section Apoptosis and necrosis. After that, the cells in each group were isolated, washed by pre-cooled 1 × PBS and lysed by 100 μL 1 × lysis buffer for 30 min at 4 °C. The lysate was centrifuged at 10,000 rpm for 10 min and the protein content in supernatant was quantified, according to a standard Bradford method. Equivalent amounts of protein extract were then incubated with colorimetric Caspase 3 substrate (Ac-DEVD-pNA) for 2 h, and OD value at 405 nm was read by an UV-vis spectrophotometer. When Caspase 3 hydrolyzes the substrate, the p-nitroaniline (pNA) moiety is released, which absorbs light specifically at 405 nm. Consequently, this experiment was based on the formation of pNA. 

#### 2.6.6. Key Markers in Mitochondrial Apoptotic Cascade

The expression of pro-apoptotic or anti-apoptotic genes, including Bax, P53, Caspase 3 and Bcl-2, that involve in mitochondrial apoptotic cascade was investigated in mRNA and protein level, on basis of classical RT-PCR and Western blot assays. [33] The procedures regarding cells seeding, cultivation and AuNPs/NIR treatment were similar to section Apoptosis and necrosis with slight modifications (cells were seeded into 6-well plate instead). For RT-PCR assay, at the end of AuNPs/NIR treatment, total RNA from each group was extracted by trizol reagent and reverse-transcribed to synthesize cDNA. Afterwards, PCR was performed to amplify gene products for P53, Bax, Bcl-2, Caspase 3 and GAPDH (internal reference), with the guidance of specific primers (Table S1). These PCR products with various lengths were separated by electrophoresis and visualized under UV lighting. Relative mRNA level of target genes were expressed as the ratio of average band density to that of GAPDH. In case of Western blot analysis, the treated cells were trypsinized and intracellular protein was extracted using pre-cooled RIPA lysis buffer containing 1 mM PMSF and 1% proteasome inhibitor. The amounts of protein in the lysate was then measured according to a standard Bradford method. After that, different protein samples from each group were loaded and electrophoresed on 10% SDS-PAGE, transferred onto a PVDF membrane and subsequently blocked with 5% skimmed milk solution for 1 h. The blots were incubated with properly diluted primary antibodies for 2 h and then with appropriate HRP-conjugated secondary antibodies for 1 h at room temperature. Protein bands were visible by a chemiluminescence reagent according to an ECL kit. The intensities of bands in each group were analyzed through Image J software. 

### 2.7. Inhibitory Activity of HA-Capped AuNPs on CSCs: Mammosphere Formation and Viability

To explore the impact of HA-capped AuNPs on self-renewable capacity of CSCs, CSC-mediated mammosphere formation was detected [34]. MDA-MB-231 cells were seeded in ultralow attachment 24-well plates at a density of 1 × 10^6^ cells per well for 24 h. After that, current complete cell culture medium was replaced with serum-free DMEM medium containing 1 × B27, 20 ng/mL EGF and 20 ng/mL bFGF, in order to screen and enrich CSCs in terms of spheroid formation. At day 7, CSCs were treated with HA-capped AuNPs at different concentrations (0, 5, 30 μg/mL), and the incubation last for 24 h. For AuNPs+L group after 20 h of AuNPs incubation, extra NIR irradiation (3 W/cm^2^ for 1 min) was applied onto these spheroids (CSCs). These NIR-irradiated CSCs were further incubated with AuNPs suspension for 4 h. CSCs without any AuNPs or NIR treatment were regarded as a control group with 100% mammosphere formation rate. At day 10, the positive mammospheres (diameter > 50 μm) from different groups were counted. In addition, positive mammospheres from each group were collected and dissociated into single CSCs, which were also counted.

To investigate the cytotoxic effect of HA-capped AuNPs towards CSCs [34], MDA-MB-231 cells were seeded in ultralow attachment 96-well plates at a density of 1 × 10^6^ cells per well for 24 h. After that, current complete cell culture medium was replaced with serum-free DMEM media containing 1 × B27, 20 ng/mL EGF and 20 ng/mL bFGF to screen and enrich CSCs. After 10 days, HA-capped AuNPs suspended in serum-free DMEM medium and a model chemotherapeutic drug, CPT, at different concentrations (0–200 μg/mL) were added into each well. CPT was first dissolved in DMSO and further diluted by serum-free DMEM before introduction to the cells. After 24 h incubation, cytotoxicity in terms of cell viability from different groups were measured based on a classical MTT reduction experiment. For AuNPs + L group, after 20 h of AuNPs incubation, extra NIR irradiation (3 W/cm^2^ for 1 min) was applied onto these spheroids (CSCs). These NIR irradiated CSCs were further incubated with AuNPs suspension for 4 h. CSCs only treated by NIR irradiation under the same condition were also studied to explore the direct impact of NIR laser on cells viability.

### 2.8. Statistics Analysis

All the experiments were performed for at least three times. Quantitative data were all expressed as mean ± SD. Statistical comparisons between two groups were determined using a two-tailed Student’s *t*-test. *p* * < 0.05, *p* ** < 0.01 and *p* *** < 0.001 were considered statistically significant or highly significant.

## 3. Results and Discussion

### 3.1. Synthesis and Characterization of HA-Capped AuNPs

In this paper, we utilized natural polysaccharide, HA, for the green synthesis of AuNPs, since different reductants always produce new types of AuNPs with unexpected physico-chemical and biological properties, including size, shape, SPR peak, anti-cancer efficiency and so on. HA is a biodegradable and biocompatible polysaccharide, which is negatively charged. The residual reductive groups in HA could reduce Au^3+^ (HAuCl_4_·3H_2_O solution, the precursor for AuNPs) to a zero-valent Au atom and subsequently generate AuNPs via Au aggregation. The polymer and negatively charged nature of HA could effectively stabilize the newly synthesized AuNPs by providing steric and static barriers. Moreover, the hydrophilic property of this HA layer would facilitate the aqueous dispersity of AuNPs. In a word, HA could be applied as a reductive, hydrophilic and capping agent. 

**Figure 1:**
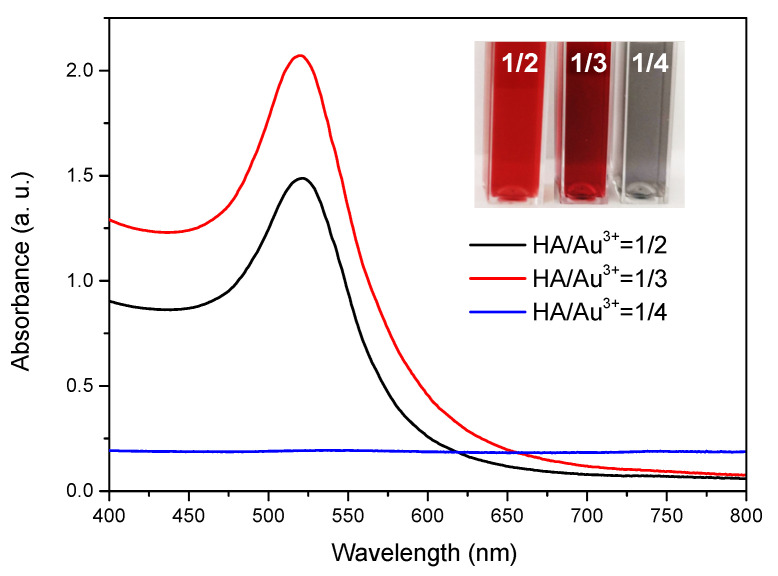
UV-vis spectra of HA-reduced AuNPs at specific HA to HAuCl_4_·3H_2_O molar ratio, reflecting characteristic SPR peaks from AuNPs. Inset, images of different batches of AuNPs suspensions.

The preparation parameters and conditions for HA-reduced AuNPs were referring to our previous research with appropriate modifications [34]. To be specific, various reductant/precursor molar ratios (HA/Au^3+^ = 1/2, 1/3, 1/4) were performed to verify the optimal preparation conditions for AuNPs. The result indicated that AuNPs at 1/2 and 1/3 HA/Au^3+^ molar ratios exhibited average particle sizes of 32.43 ± 0.23 nm and 27.36 ± 0.32 nm, respectively (Table 1). In contrast, AuNPs at 1/4 molar ratio showed significantly greater particle size (300.20 ± 7.32 nm) and PDI value (0.312 ± 0.012), which implied the possible aggregated and poly-dispersed particles. The appearance of ruby/dark red uniform AuNPs suspension and corresponding SPR peaks at 520 nm were clearly observed at 1/2 and 1/3 HA/Au^3+^ ratio (Figure 1), validating the successful formation of AuNPs under that condition. In comparison, dark grey suspension was observed for 1/4 AuNPs, which yielded no SPR peak. It was probably because reductive HA was not sufficient to stabilize AuNPs, which caused their aggregation and subsequent precipitation. In consequence, 1/4 HA/Au^3+^ ratio was first excluded. 

**Figure 2:**
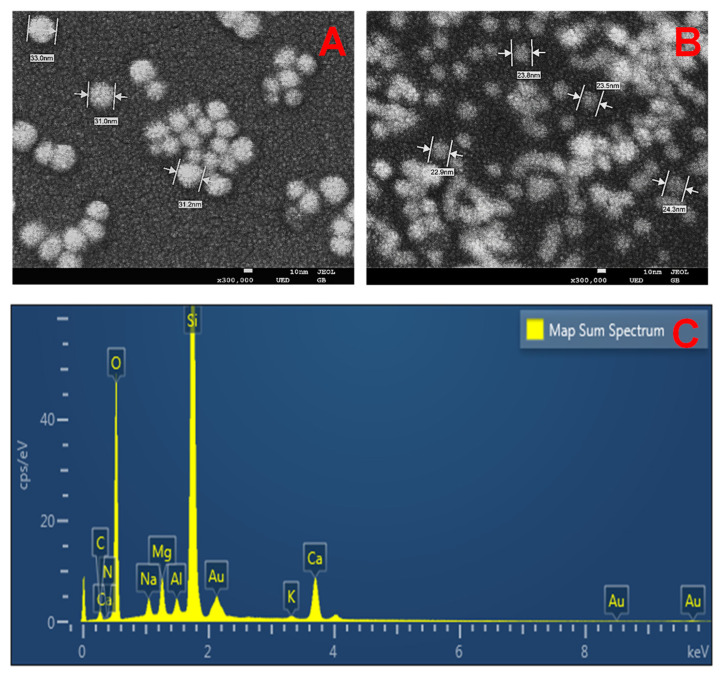
SEM images of HA-reduced AuNPs at 1/2 (**A**) and 1/3 (**B**) HA/Au^3+^ molar ratios. Scale bar = 10 nm. (**C**): EDX spectrum of HA-reduced AuNPs at 1/2 HA/Au^3+^ molar ratio.

For 1/2 and 1/3 AuNPs, they shared similar small particle sizes and high negative zeta potentials (−45.68 ± 0.79 and −42.05 ± 1.21 mV), reflecting good colloidal stability by the negative HA layer (Table 1). Although 1/3 HA/Au^3+^ ratio resulted in higher yield of AuNPs in terms of more intense SPR peak (Figure 1), they displayed greater PDI value than 1/2 AuNPs (0.291 ± 0.030 vs. 0.057 ± 0.005), indicating poly-dispersed AuNPs. This result was further confirmed by SEM imaging. As Figure 2A depicted, 1/2 AuNPs demonstrated mono-dispersed spherical particles, with uniform diameters of approximate 31 nm. On the contrary, a mixture of particles in different shapes, such as rod and spheres, were observed in 1/3 AuNPs (Figure 2B) and they were poly-dispersed with a wide size range. As with previously reported literature [35], AuNPs with spherical structure, 20–30 nm in size and with uniform distribution are always advantageous for photothermal efficiency, intracellular uptake, anti-cancer activity and facile excretion by renal clearance. In consequence, HA-capped AuNPs at 1/2 HA/Au^3+^ molar ratio were selected for further research. From Figure 2C, EDX map chart disclosed the strong signal of Au, indicating the formation and high purity of synthesized AuNPs. In addition, carbon, nitrogen and oxygen peaks with different intensity signals were also present, originating from HA layer. The yield rate of Au for 1/2 AuNPs was calculated to be 76.2%. 

**Figure 3:**
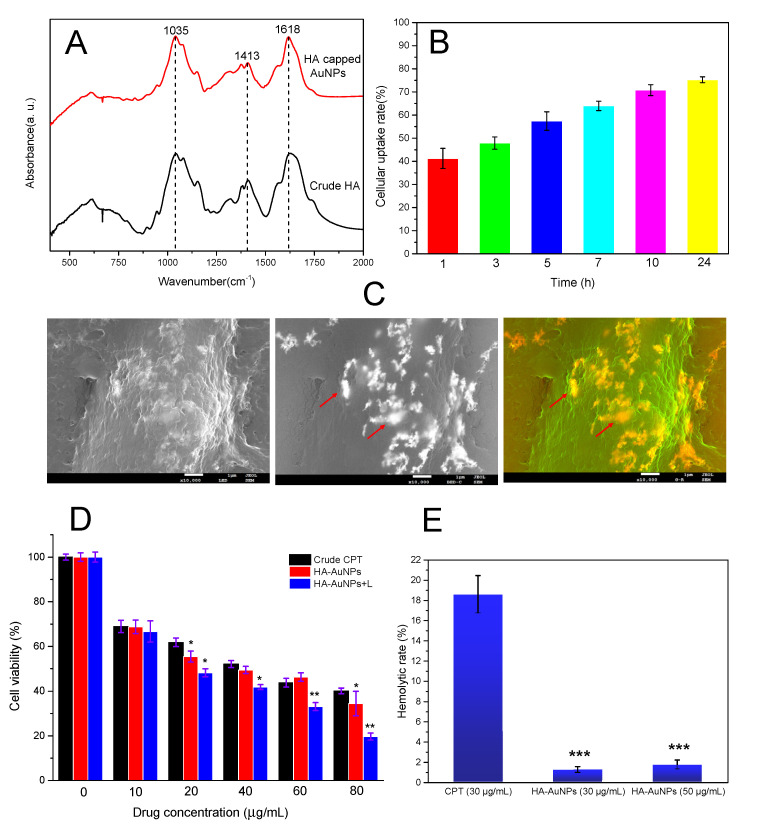
(**A**) FTIR absorption spectra of HA-capped AuNPs together with crude HA powder. (**B**) Cellular uptake efficiency of HA-capped AuNPs (Conc. =20 µg/mL) at different incubation time in MDA-MB-231 cells. (**C**) Representative SEM images showing the intracellular AuNPs in MDA-MB-231 cells after 24 h incubation, arrows emphasize the existence of AuNPs aggregates inside the cells. (**D**) Inhibitory effect of different drugs at various concentration against bulk MDA-MB-231 cells. (**E**) Hemolytic effect of HA-capped AuNPs or CPT under different working concentrations, reflecting their respective hemocompatibility. * p < 0.05, ** p < 0.01 and *** p < 0.001 vs. CPT group.

FTIR analysis could provide direct proof for the successful synthesis of HA-capped AuNPs. Although AuNPs did not respond well to FTIR, HA did. The FTIR spectra of dried HA-capped AuNPs together with pure HA powder was shown and compared in Figure 3A. In general, HA-capped AuNPs demonstrated nearly the same absorption profile as crude HA. The band at 1618 cm^−1^, corresponding to stretching of C=O in COO^−^ group was observed in both samples. However, this typical peak in the HA-capped AuNPs spectrum was sharper than that in HA spectrum, resulting from the interaction (such as metal coordination) between AuNPs and HA. Moreover, the other characteristic HA absorption peaks at 1035 cm^−1^, assigned to C-N stretching; 1372 cm^−1^, assigned to C-H stretching; 1413 cm^−1^, corresponding to amide I band, were all present in both samples. The result validated the existence of HA as both a reductant and stabilizer for newly produced AuNPs. 

To evaluate the storage stability of HA-capped AuNPs, their intensity of SPR peak and aqueous dispersity were monitored under different storage temperature and time. As Figure S1A,B showed, no significant decrease in SPR peak intensity was observed at 4 °C or room temperature within a period of 12 days, proving good storage stability of HA-capped AuNPs. When it was up to 15 days, a slight drop in SPR peak intensity occurred under room temperature, which might result from a small proportion of precipitated AuNPs. Theoretically, the hydrophobic nature and high Gibbs free energy of metal nanoparticles drives them to aggregate and settle down. On the contrary, there was still no change in SPR spectrum for AuNPs suspension at 4 °C because molar thermal motion would slow down under low temperature, thus decreasing the possibility of aggregates or agglomerates of AuNPs. From Figure S1C, HA-capped AuNPs could disperse well into water and form a uniform colloid suspension at 4 °C or room temperature, and there was no remarkable variation in morphology of the suspension even after 15 days. Not as expected, precipitated AuNPs were not observed by naked eyes. Although no apparent sedimentation and precipitation occurred, the lower SPR intensity of AuNPs at room temperature implied the formation of larger aggregates. Since sedimentation is highly dependent on particle size, these aggregated AuNPs are still not large enough to settle down. All the results supported the good aqueous dispersity and storage stability of HA-capped AuNPs, attributing from the negatively charged HA layer. HA layer not only increased the hydrophilicity of AuNPs but also provided static and steric barriers to prevent aggregation.

Hyperthermia therapy is an excellent non-invasive and controllable cancer treatment strategy, in which cancer cells would be destroyed via DNA fragmentation, protein denaturation and membrane lysis under fatal temperatures (above 43 °C). Cancer cells are more sensitive towards heat than healthy cells, due to their abnormal structure and metabolism. In a word, hyperthermia therapy is highly selective to the extent that it could differentiate between cancer cells and normal ones. AuNPs have been widely applied as a good photothermal agent after exposure to the energy sources, such as NIR. NIR could penetrate deep tissues, since it is nearly transparent for the protein and liquid in the body. In consequence, the photothermal effect of HA-capped AuNPs was examined before their bioactivity study, hoping to verify their potential on eradiation of both bulky cancer cells and CSCs. 

The temperature variations of HA-capped AuNPs suspensions (Conc. = 0–50 µg/mL) exposure to NIR irradiation was monitored at different power densities and durations (Figure S2). Generally, AuNPs suspension under all the conditions underwent a faster temperature increment and higher equilibrium temperature than the negative control group (pure cell culture medium). The degree of temperature rise was positively related with concentration of HA-capped AuNPs, laser power density and lighting time. When irradiation time was fixed at 10 min, the ultimate temperature of AuNPs (10 µg/mL) suspension were 33, 47 and 50 °C upon an irradiation power of 1, 2 or 3 W/cm^2^, respectively. In contrast, when irradiation power was fixed at 1 W/cm^2^, the equilibrium temperature varied from 33°C to 41 °C, as AuNPs concentrations rose from 10 to 50 µg/mL after 10 min lighting. These data indicated that NIR power density played a significant role on photothermal effect of HA-capped AuNPs, therefore, 3 W/cm^2^ was set for the future research. From Figure S2C, the temperature of suspension rapidly increased from 25 °C to 51 °C within only 1 min at maximum AuNPs concentration (50 µg/mL). When the concentration of AuNPs dropped to 10 µg/mL, the temperature still reached 42 °C, which was basically sufficient to cause cancer cells death. In contrast, less temperature change (25 °C to 31 °C) was observed for the control group upon the equal NIR treatment (3 W/cm^2^, 1 min). Overall, the laser treatment conditions of 3 W/cm^2^ and 1 min were desired, since the generated heat for hyperthermia therapy could be simply controlled by adjusting AuNPs concentrations. In other words, the high anti-cancer activity against bulk cancer cells and CSCs and low injury on normal cells could be rationally balanced under that condition. 

### 3.2. Level of Cellular Uptake of HA-Capped AuNPs in MDA-MB-231 Cells 

In order to acquire a perfect therapeutic efficiency of HA-capped AuNPs, a high cellular uptake rate is expected. As is well known that intracellular transportation of the chemotherapeutic drugs is closely associated with ultimate anti-cancer outcome. In this study, the drug internalization process was investigated by a quantitative assay and SEM observation. From Figure 3B, HA-capped AuNPs demonstrated a rapid intracellular transportation—more than 40% were taken up within only 1 h and up to over 75% at 24 h. In general, the cellular uptake of AuNPs conformed to a time-dependent manner. The high drug delivery efficiency is largely due to their nano-scaled nature, since nanoparticles could be quickly endocytosed via caveolae- or clathrin-mediated pathways, avoiding pumping out from cells by ABC transporters on surface of cancer cells [36]. It was reported that citrate-reduced AuNPs relied on such an endocytosis process in macrophage cells [37]. Moreover, HA layer could specially recognize and interact with over-expressed CD44 receptors on surface of cancer cells, which could trigger a ligand-receptor-mediated endocytosis, thus adding benefits to the drug delivery efficiency. As predicted, the localization and distribution of abundant HA-capped AuNPs were observed inside MDA-MB-231 cells from the SEM images (Figure 3C). A proportion of HA-capped AuNPs formed aggregates, probably because the HA layer would undergo protonation under mild acid tumor microenvironment, thus neutralizing their negative charges and weakening their stabilizing function. The surface refractive index of AuNPs aggregates would be different from the dispersed counterparts, particularly the resulting aggregates with larger sizes would be red shifted from visible light (520 nm) to NIR region, leading to stronger SPR absorption and subsequent photothermal effect. In a word, the intracellular AuNPs aggregates were favorable for cancer treatment. 

### 3.3. In Vitro Anti-Cancer Activity, Selectivity and Hemolytic Effect of HA-Capped AuNPs

The cytotoxic effect of HA-capped AuNPs and a model chemotherapeutic drug, CPT, was studied on breast cancer cell line MDA-MB-231 and normal L929 cells. The cells were treated for 24 h at various concentrations ranging from 0 to 80 µg/mL and relevant IC_50_ values were determined. From Figure 3D, the first blue column at drug concentration of 0 µg/mL represented MDA-MB-231 cells treated with NIR only. As could be observed, the viability of MDA-MB-231 cells still reached almost 100% after NIR irradiation. Thus, the effect of NIR laser on cell viability should be excluded. As Figure 3D and Table 2 depicted, bulk MDA-MB-231 cells demonstrated a significant resistant effect against CPT: MDA-MB-231 cells showed almost 61.7% viability even at high CPT concentration (20 µg/mL) and IC_50_ value was 36.9 ± 1.6 µg/mL. This is a common phenomenon for most organic anti-cancer agents because they always show poor water solubility, low bioavailability and instability, leading to inefficient chemotherapy. Most importantly, cancer cells would develop specific chemo-resistance after frequent exposure to the single drugs. One of possible causes of drug resistance is overexpression of the P-glycoprotein transporter (Pgp), which is coded by *MDR1* gene. Pgp is an energy-dependent drug efflux pump, alleviating the cytotoxic effect of current drugs. Actually, MDA-MB-231 cells in this study demonstrated a high expression level in the *MDR1* gene (data not shown). That is why we explore new anti-cancer agents or treatment modes except chemotherapy. By contrast, improved cytotoxicity could be observed when MDA-MB-231 cells were treated with HA-capped AuNPs. In general, HA-capped AuNPs reduced the viabilities of MDA-MB-231 cells in a dose-dependent manner. Cell viability rate ranged from 68.6% to 34.1% at 10 to 80 µg/mL for HA-capped AuNPs and the resultant IC_50_ value was 34.8 ± 1.8 µg/mL. To investigate the combined effect from AuNPs and their photothermal activity upon NIR laser treatment, the anti-cancer effect of HA-capped AuNPs in the presence of NIR lighting was monitored under the same conditions. As predicted, the dual treatments (chemotherapy + hyperthermia therapy) resulted in highest cytotoxicity and the IC_50_ value turned out to be 22.4 ± 1.4 µg/mL, implying the notable tumor ablation from the extra photothermal effect of AuNPs. Except for that, the inherent anti-cancer capacity, nano-scaled structure and high cellular uptake efficiency of HA-capped AuNPs also contributed to their ultimate excellent anti-cancer activity. 

For clinical chemotherapeutic agent, their potential toxic effect towards normal cells should be also evaluated. As Table 2 indicated, CPT exhibited a remarkable toxic effect against L929 cells in terms of lowest IC_50_ value (15.9 ± 1.3 µg/mL). This is why their clinical application is postponed unless there is proper modification. In comparison, HA-capped AuNPs with/without NIR lighting both exhibited much lower cytotoxicity in terms of higher IC_50_ values. Regarding the strong anti-cancer activity, AuNPs with/without NIR lighting demonstrated excellent biosafety, that is, they could discriminate between cancer cells and normal cells. This feature was owing to green synthesis of AuNPs, inherent biocompatibility of AuNPs and higher heat tolerance of normal cells, as compared to cancer cells [16,17,38].

To confirm the biocompatibility of HA-capped AuNPs, their hemocompatibility was also assessed based on a hemolysis assay, since most chemotherapeutic drugs would be preferentially administered via intravenous route. As Figure 3E depicted, crude CPT caused over 18.6% hemolytic rate at 30 µg/mL, indicative of their deleterious effect on erythrocytes during circulation. In contrast, HA-capped AuNPs at approximate IC_50_ or high concentration displayed negligible hemolytic rates (less than 2%), within the threshold of hemocompatibility. This result was probably due to green synthesis of AuNPs and their inherent biocompatibility. Unlike flexible and hydrophobic CPT, the rigid nature of AuNPs and negative HA layer also helped prevent their attachment to erythrocytes. In sum, HA-capped AuNPs demonstrated excellent anti-cancer activity, selection and biosafety. 

### 3.4. Apoptotic Activity of HA-Capped AuNPs 

As was widely reported, nanomaterials could always induce a couple of death modes including autophagy, apoptosis and necrosis in cancer cells, all of which contribute to their overall improved cytotoxicity. For example, chemical- or physical-synthesized AuNPs could trigger apoptosis, damage cell nucleus, induce cell cycle arrest and prevent cell division [39,40]. To further validate the mechanism of the cytotoxic activity of HA-capped AuNPs, apoptosis and necrosis of MDA-MB-231 cells were studied. 

**Figure 4:**
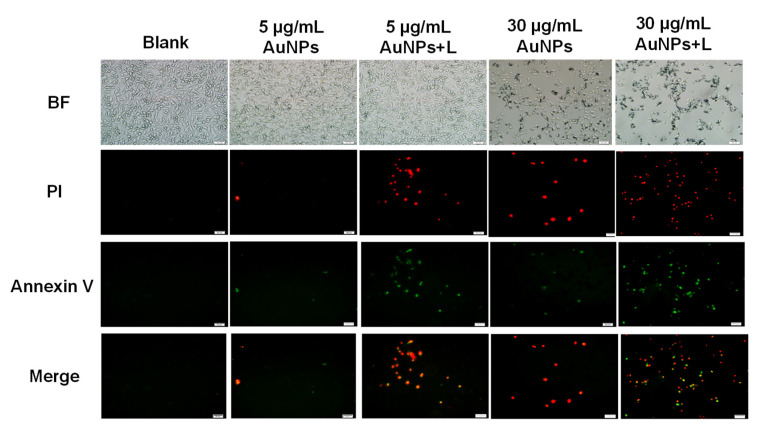
Apoptosis and necrosis in MDA-MB-231 cells upon exposure to HA-capped AuNPs (with/without NIR lighting) at different Au concentrations, according to an AnnexinV/PI double staining assay. Scale bar represents 50 μm.

The apoptotic or necrotic activity of HA-capped AuNPs was evaluated in MDA-MB-231 cells by an AnnexinV-FITC/PI staining method. The representative areas demonstrating apoptosis or necrosis from each group were shown in Figure 4. In this study, MDA-MB-231 cells with green or yellow fluorescence (AnnexinV-FITC or AnnexinV-FITC/PI positive) represented early or advanced apoptotic cells and those with red color were regarded as necrotic cells, since they lost the integral membrane, and thus would be stained by PI only. From Figure 4, after treatment with low concentration of HA-capped AuNPs (5 µg/mL), MDA-MB-231 cells presented slight apoptosis and necrosis in terms of little distributed fluorescence. When NIR irradiation was applied, remarkable increase in the proportion of fluorescent cells was observed, reflecting improved apoptotic or necrotic activity. Therefore, hyperthermia played a vital role on apoptosis induction under low AuNPs concentration; in other words, the chemotherapeutic effect was covered by the photothermal effect from AuNPs. In accordance with our expectation, less than 50% MDA-MB-231 cells survived after exposure to approximate IC_50_ concentration (30 µg/mL) of HA-capped AuNPs (image photographed under bright field). Among the remaining living cells, the total apoptotic and necrotic ratio was approximately 10% for 30 µg/mL AuNPs treated group and 50% for 30 µg/mL AuNPs + L group. It would be tentatively concluded: (1) except for cytotoxic effect of HA-capped AuNPs, their apoptotic and necrotic activity also accounted for the ultimate death mode of bulk MDA-MB-231 cells; (2) as the concentration of AuNPs increased, their cytotoxic effect contributed more and more in killing cancer cells; (3) the combination of chemotherapy and hyperthermia therapy resulted in more severe apoptosis and necrosis events. 

### 3.5. The Morphology of Nucleus and Integrity of Plasma Membrane

**Figure 5:**
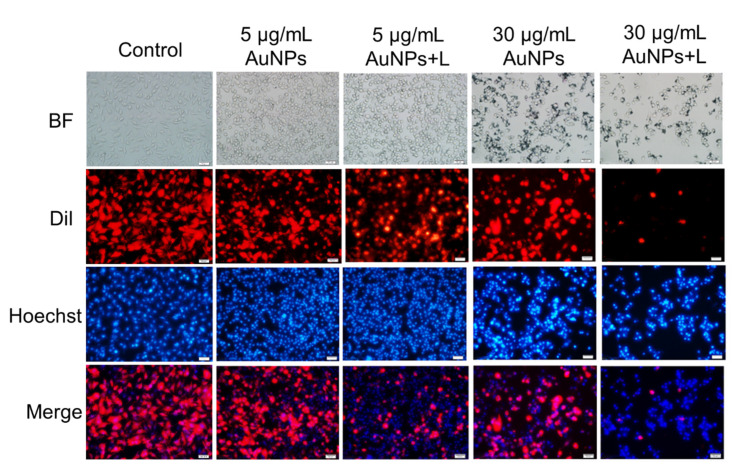
The change in morphology of nucleus and plasma membrane in MDA-MB-231 cells upon exposure to HA-capped AuNPs (with/without NIR lighting) at different Au concentrations, according to a Hoechst/DiI double staining assay. Scale bar represents 50 μm.

Damage in cell nucleus and a loss in plasma membrane integrity are also key features of apoptosis or necrosis. To further determine the apoptosis induction capacity of HA-capped AuNPs, Hoechst and DiI staining was carried out to detect nuclear and membrane damage in MDA-MB-231 cells. As Figure 5 showed, control MDA-MB-231 cells presented integral plasma membranes with bright red fluorescence and uniform blue fluorescent nuclei. There was an insignificant change in morphology of nucleus and cell membranes after exposure to 5 µg/mL HA-capped AuNPs, implying limited apoptotic or necrotic effect. By contrast, for 5 µg/mL AuNPs + L group, partial MDA-MB-231 cells lost intactness in membrane, reflected by the less distributed red fluorescence. Consistent with AnnexinV/PI staining result, photothermal effect from AuNPs played a more important role at low AuNPs concentration, and the generated heat caused cell membrane lysis via increasing its permeability or directly destroying the structure. As the concentration of AuNPs increased to IC_50_ scale, a majority of cell membranes underwent shedding, deformation or complete damage. In addition, this group elicited significant variation in nucleus morphology in MDA-MB-231 cells, to be specific, many dark stained nuclei with brighter fluorescence were observed, resulting from condensed chromosome or apoptosis bodies. As expected, the 30 µg/mL AuNPs + L group induced the most pronounced apoptotic and necrotic event—most plasma membranes disappeared due to severe impairment and a greater proportion of dark stained cell nucleus were observed. All the results verified apoptotic and necrotic activity from HA-capped AuNPs, especially in combination with NIR irradiation.

### 3.6. Mitochondrial Apoptotic Pathway

Mitochondria play a vital role in energy supply, apoptosis triggering and maintenance of cell structure and function. It was previously reported that a couple of chemotherapeutic drugs, metal nanoparticles or other cytotoxic reagents would target mitochondria to cause their dysfunction. The damage of mitochondria is always reflected in both structure and function (a loss in MMP or halted functioning of electron transport chain), which are responsible for subsequent metabolic outcomes, such as the high level of mitochondrial ROS, oxidative DNA fragmentation, activation of mitochondria-derived caspases and increased/decreased expression of pro-apoptotic/anti-apoptotic genes. These physiological changes are always early events for mitochondria-mediated apoptosis. In consequence, mitochondrial apoptotic activity of HA-capped AuNPs was explored according to a series of experiments, such as ROS level, mitochondria morphology and MMP, Caspase 3 activation and expression of key genes that modulate mitochondrial apoptotic pathway. 

**Figure 6:**
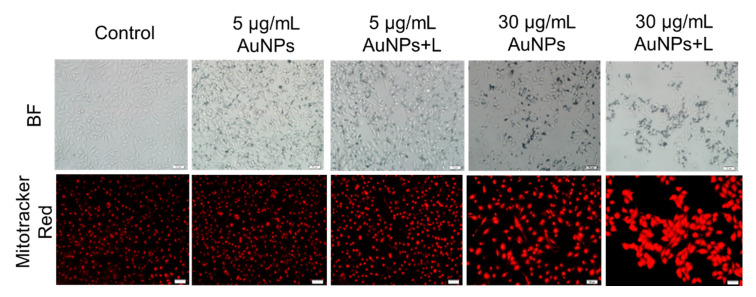
Morphology of mitochondria in MDA-MB-231 cells upon exposure to HA-capped AuNPs (with/without NIR lighting) at different Au concentrations, according to a Mitotracker Red staining assay. Scale bar represents 50 μm.

First, Mitotracker Red and JC-1 staining were performed to monitor the change in morphology and MMP of mitochondria in MDA-MB-231 cells, upon exposure to HA-capped AuNPs at different concentrations. From Figure 6, there are a considerable number of mitochondria inside untreated MDA-MB-231 cells, due to high energy provide for rapidly dividing cancer cells. Normal mitochondria were observed with nearly uniform sizes, around 10 µm in long axis. No apparent variation in mitochondria morphology occurred after treatment with low concentration of HA-capped AuNPs, regardless of NIR treatment or not. In contrast, both 30 µg/mL AuNPs group and 30 µg/mL AuNPs + L group induced significant damage in mitochondria in terms of lower number, deformation and swelling. To be specific, in 30 µg/mL AuNPs + L group, most mitochondria swelled and the sizes increased up to over 25 µm, a typical characteristic of depolarization. Therefore, a change in MMP due to mitochondria dysfunction was further examined. In Figure S3, healthy mitochondria in control group displayed high MMP with complete polarization. Here, JC-1 indicator would form aggregates with strong red fluorescence. On the contrary, apoptotic cells demonstrated a decrease in MMP, so JC-1 would keep the monomeric form, thus presenting green fluorescence instead. For 5 µg/mL AuNPs group, the high proportion of JC-1 aggregates indicated no significant loss in MMP. By contrast, increased percentage of JC-1 monomers were found in 5 µg/mL AuNPs plus NIR laser group. This change in fluorescence pattern reflected that combination of hyperthermia and AuNPs caused depolarization of mitochondria membrane to some extent. Moreover, as the concentration of AuNPs increased up to IC_50_ scale, dramatic decreased number of mitochondria was observed due to strong cytotoxicity. Among the remaining mitochondria, the proportion of green fluorescence increased regardless of NIR treatment: the ratio of JC-1 monomers in these two groups were estimated to be more than 70%. These two experiments confirmed that HA-capped AuNPs would alter the functional status of mitochondria. They could induce mitochondria-mediated apoptosis and the increment in concentration or introduction of NIR laser irradiation would dramatically enhance this impact. It could be tentatively deduced that HA-capped AuNPs could directly or indirectly target mitochondria to the benefit of their excellent cytotoxicity and apoptosis induction efficiency. 

**Figure 7:**
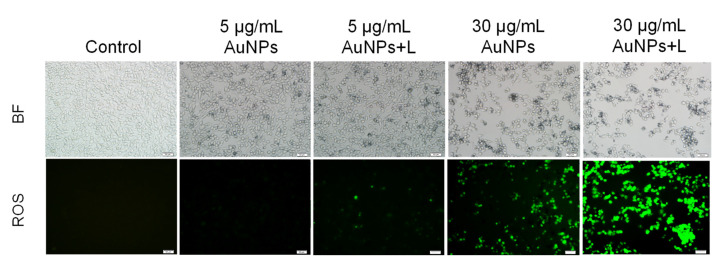
Generated ROS in MDA-MB-231 cells upon exposure to HA-capped AuNPs (with/without NIR lighting) at different Au concentrations, according to a DCFH-DA staining assay. Scale bar represents 50 μm.

Dysfunction in mitochondria is closely related with excessive ROS release, and then promotes apoptosis progress. In turn, ROS-mediated oxidative stress would further aggravate the damage of mitochondrial membrane. To investigate possible oxidative stress induced by HA-capped AuNPs, intracellular ROS level was measured in MDA-MB-231 cells based on a DCFH-DA loading method. As Figure 7 showed, negative control and 5 μg/mL AuNPs group displayed little green fluorescence, suggesting low level of generated ROS. This result agree well with previous assay. On the other side, 5 μg/mL AuNPs + L group induced more ROS generation, implying greater susceptibility to oxidative stress. As the concentration of AuNPs increased to IC_50_ scale, both AuNPs and AuNPs + L group demonstrated pronounced fluorescence intensity from increased ROS level, and the latter group led to more significant results. In general, ROS level was positively proportional to the concentration of HA-capped AuNPs and the introduction of photothermal effect. The high ROS generation would unbalance the normal redox homeostasis of MDA-MB-231 cells, subsequently destroying DNA and other biomolecules and activated caspases, contributing to the execution of mitochondrial apoptosis. 

**Figure 8:**
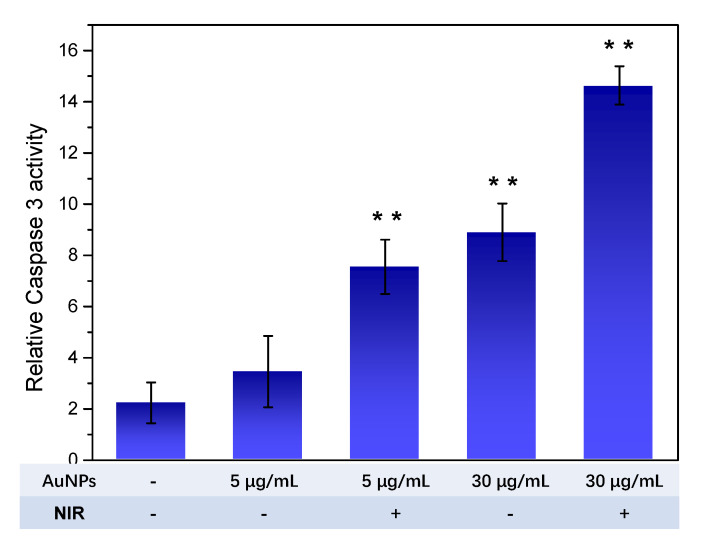
Relative Caspase 3 activity of MDA-MB-231 cells upon exposure to HA-capped AuNPs (with/without NIR treatment) at different concentration. ** *p* < 0.01 vs. negative control group.

Caspase 3, which belongs to the caspases family, is involved in intrinsic mitochondria-mediated apoptosis. It is activated by specific pro-apoptotic factors, including cytochrome c and ROS from depolarized mitochondria. The induction of Caspase 3 means that apoptosis is imperative and could be no longer reversed. Therefore, the effect of HA-capped AuNPs on the activation level of Caspase 3 was measured. As Figure 8 illustrated, in comparison with negative control cells, MDA-MB-231 cells treated with HA-capped AuNPs with/without NIR laser lighting all showed improved Caspase 3 activation level at varying degrees. Similarly, HA-capped AuNPs (at IC_50_ concentration) in the presence of NIR irradiation resulted in the highest Caspase 3 level, almost 6.5-fold higher than untreated cells. The result indicated that a loss in integrity of mitochondria, caused by AuNPs plus NIR laser, promoted the activation of Caspase 3, contributing to cell death via apoptosis.

Apoptosis is regulated through upregulation of some pro-apoptotic genes, such as *p53*, *Bax*, *Caspases,* and to a lesser extent in anti-apoptotic genes, such as *Bcl-2*. These genes would be turned on/off in response to specific signals, such as damaged mitochondria, determining the apoptosis or survival of cancer cells. For example, p53 protein could positively regulate the development of apoptosis by inducing the transcription of *Bax*, which would counteract the function of anti-apoptotic protein Bcl-2. Moreover, an imbalance between Bax and Bcl-2 activates Caspase 3-dependent apoptosis. In this study, the impact of HA-capped AuNPs on expression level of key apoptotic markers, such as *p53*, *Bax*, *Bcl-2* and *Caspase 3*, were monitored in MDA-MB-231 cells by RT-PCR and Western blot analysis. 

**Figure 9:**
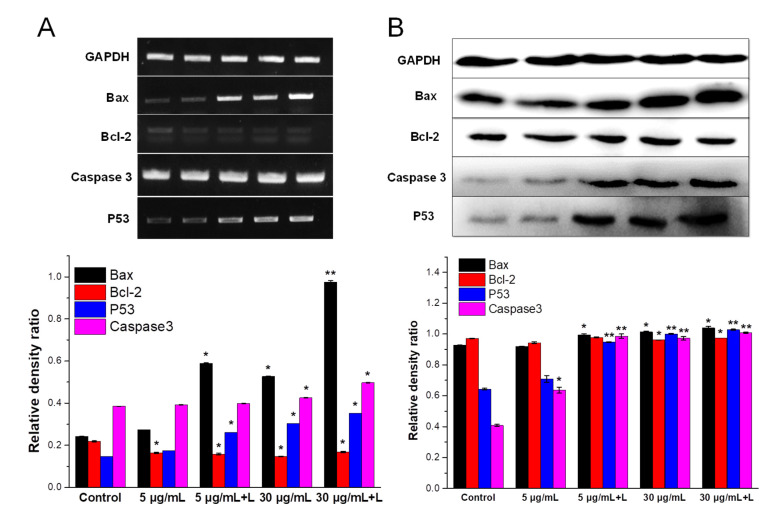
Effect of HA-capped AuNPs with/without NIR treatment on expression of apoptosis-related genes (*Bax*, *Bcl-2*, *P53* and *Caspase 3*) in MDA-MB-231 cells, according to RT-PCR (**A**) and Western blot (**B**) assay. Relative mRNA or protein expression level for each gene were all normalized by internal control *GAPDH* and shown in respective bar graphs. * p < 0.05, ** p < 0.01 vs. control group.

For RT-PCR result (Figure 9A), all the groups indicated increased P53 expression, which regulates the transcription of Bax and Bcl-2. As a result, the mRNA level of Bax and Caspase 3 in all treated groups were remarkably higher than control group, whereas there was a decrease in Bcl-2 level, causing an imbalance between Bax and Bcl-2. As predicted, 30 μg/mL AuNPs + L group induced the highest level of P53, Caspase 3 and Bax to Bcl-2 ratio, thus increasing the severity of mitochondrial apoptosis. Western blot assay further verified the lower protein expression of Bcl-2 and corresponding upregulation in Bax, P53 and Caspase 3, after exposure to 30 μg/mL AuNPs plus NIR treatment (Figure 9B). Consistent with previous results (Figure 8), elevated activation level of Caspase 3 by HA-capped AuNPs was partially related to their increased protein expression. 

In sum, our findings confirmed that HA-capped AuNPs together with NIR treatment triggered cancer cell apoptosis via an intrinsic mitochondrial apoptotic pathway, in terms of destructive mitochondria, depolarization of mitochondrial membrane, oxidative stress and activation of Caspase 3 in MDA-MB-231 cells. The mechanistic relationship and molecular targets were closely related with upregulation in *P53*, *Bax* and *Caspase 3* genes and downregulation in *Bcl-2* gene. The excellent apoptosis induction capacity of HA-capped AuNPs contributed to the overall cytotoxicity against bulk cancer cells, based on a smart combination of AuNPs and their photothermal effect. 

### 3.7. Inhibitory Activity of HA-Capped AuNPs on CSC: Mammosphere Formation Capacity and CSC Viability

Constant self-renewal could keep the undifferentiated condition of CSCs, contributing to the severe resistance towards chemotherapy. To evaluate the impact of HA-capped AuNPs on self-renewable capacity and viability of MDA-MB-231 CSCs, a mammosphere formation assay and a MTT experiment were both performed, respectively [27].

MDA-MB-231 CSCs were isolated, identified and enriched according to mammospheres formation screening method. Under certain (non-adherent, serum-free) cultivation conditions, CSCs, but not bulk MDA-MB-231 cells, are able to survive and form a spheroid called mammosphere. These enriched CSCs were further identified by measuring the expression level of pluripotency related genes by a RT-PCR analysis. From Figure S4, the transcriptional abundance of stemness-related genes (*Nanog* and *Sox-2*) in MDA-MB-231 CSCs were much higher than that in bulk MDA-MB-231 cells, confirming the successful enrichment of CSCs. 

**Figure 10:**
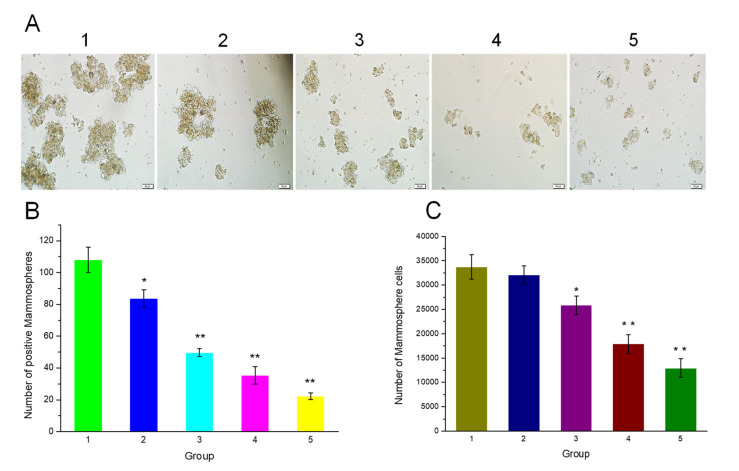
(**A**) The morphology of mammospheres after treatment with HA-capped AuNPs (with or without NIR laser) at different concentrations, scale bar = 50 μm. The number of surviving mammospheres (**B**) or isolated mammosphere cells (**C**) after treatment with HA-capped AuNPs (with or without NIR laser) at different concentrations. The numbers represent the following: 1 for control group, 2 for 5 μg/mL AuNPs group, 3 for 5 μg/mL AuNPs + L group, 4 for 30 μg/mL AuNPs group, 5 for 30 μg/mL AuNPs + L group. * p < 0.05, ** p < 0.01 vs. control group.

As Figure 10A showed, compared with the control group, a remarkable decrease in the average sizes of mammospheres was observed in all treated groups. As expected, the 30 µg/mL AuNPs + L group demonstrated the most significant result, from 150 µm (Control group) to 50 µm. Moreover, the surviving positive mammospheres from each group were also counted after AuNPs/NIR treatment (Figure 10B). The treated groups showed a 22.5%, 54.0%, 67.2% and 79.3% reduction in positive mammosphere number when treated with 5 µg/mL AuNPs, 5 µg/mL AuNPs plus NIR irradiation, 30 µg/mL AuNPs and 30 µg/mL AuNPs plus NIR irradiation, respectively. The number of mammosphere cells of each group was also measured (Figure 10C). Similarly, 30 µg/mL AuNPs plus NIR irradiation resulted in the minimum number. These results indicated that the combination of AuNPs at higher concentration with their elicited photothermal effect exhibited maximum inhibitory impact on the self-renewable capacity of CSCs.

As Figure S5 and Table 2 depicted, NIR irradiation itself failed to pose any negative impact on cell viability: CSCs displayed nearly 100% viability after treatment of NIR only under the same conditions (first light green column). On the other side, the inhibitory effect of HA-capped AuNPs on CSCs viability was much greater than free CPT. This result was consistent with the fact that CSCs showed strong resistance against a variety of chemotherapeutic agents, such as CPT. To be specific, elimination of CSCs by HA-capped AuNPs with or without NIR treatment complied with a concentration-dependent manner. Consistent with previous data, HA-capped AuNPs plus NIR treatment led to lowest CSC viability with IC_50_ value of 97.7 ± 5.6 µg/mL, suggesting a synergistic effect between HA-capped AuNPs and hyperthermia. It was understandable that bulk MDA-MB-231 cells were more sensitive to HA-capped AuNPs than CSCs subpopulation: relevant IC_50_ value was approximately 4-fold higher for CSCs than bulk cancer cells (Table 2), which was determined by the stubborn resistance nature of CSCs. Nevertheless, HA-capped AuNPs are still advantageous on eliminating CSCs, as compared to other organic anti-cancer drug. In sum, HA-capped AuNPs plus NIR irradiation would dramatically enhance therapeutic efficacy towards both bulk MDA-MB-231 cells and CSCs. 

Based on all the results, HA-capped AuNPs, especially in combination with NIR irradiation, could highly differentiate between bulk cancer cells/CSCs and normal cells, that is, they were more toxic against cancer cells than normal ones. This phenomenon was attributed from the intrinsic anti-cancer activity from AuNPs, over-expressed CD44 receptors on surface of bulk cancer cells/CSCs, different hyperthermia tolerance between cancer cells and normal cells, green synthesis and inherent biocompatibility of AuNPs. To be specific, HA could specifically recognize and bind to CD44 receptors, which are overexpressed on the surface of CSCs and bulk cancer cells, but barely expressed on normal cells, so more HA-capped AuNPs could be transported into cancer cells, leading to greater cytotoxicity. On the other side, AuNPs demonstrated their anti-cancer effect via various actions, such as DNA damage, interference in cell cycles, inhibition in thioredoxin reductase and proteasome and regulation in specific kinases. These multiple modes of action accounted for the improved anti-cancer activity, especially against drug-resistant CSCs. Moreover, the excellent photothermal performance from AuNPs upon exposure to NIR laser also contributed to the selective toxicity, because cancer cells were more sensitive to hyperthermia than healthy cells due to their more vigorous metabolic activity and difficulty in dissipating heat from the abnormal structure. Finally, the green synthesis and biocompatibility nature of AuNPs also resulted in their low cytotoxicity towards normal cells.

## 4. Conclusions

For combination of both chemotherapy and hyperthermia therapy, HA functionalized AuNPs were fabricated in a green and simple way, to effectively improve overall anti-cancer effect towards both bulk cancer cells and CSCs, and meanwhile, alleviate side/toxic effect. Taking advantage of versatile HA layer and nanosized structure, these optimized AuNPs demonstrated excellent physical characteristics and high cellular uptake rate, contributing to a strong inhibitory effect on both bulk MDA-MB-231 cells and CSCs. In particular, in comparison with chemotherapy alone, the cooperative chemo-photothermal therapy of HA-capped AuNPs plus NIR irradiation demonstrated more potent therapeutic outcomes in terms of cytotoxicity, apoptosis and necrosis. Mitochondria-dependent intracellular cascade was involved in the apoptosis process in treated MDA-MB-231 cells; the morphological change of mitochondria; a loss in MMP; damaged cell nucleus and plasma membrane; and increased ROS release and intense Caspase 3 activity. In addition, HA-capped AuNPs-induced apoptotic responses included an increase or decreased level in *Bax*, *P53*, *Bcl-2* and *Caspase 3* at gene and protein level. Considering the anti-cancer potential and biocompatibility, HA-capped AuNPs are expected to be an alternative chemotherapeutic drug to treat breast cancer, with lower possibility of CSC-caused recurrence and metastasis. Although more research, such as in vivo study, is needed, the promising aspects of this new drug candidate could be seen. 

## Figures and Tables

**Table 1:** Average particle size, PDI value and zeta potentials of different HA-reduced AuNPs.

HA/Au^3+^	Particle Sizes (nm)	PDI	Zeta-Potential (mV)
1/2	32.43 ± 0.23	0.057 ± 0.005	−45.68 ± 0.79
1/3	27.36 ± 0.32	0.291 ± 0.030	−42.05 ± 1.21
1/4	300.20 ± 7.32	0.312 ± 0.012	n.a.

**Table 2:** IC_50_ values (µg/mL) of different samples against different types of cells.

Samples	Bulk MDA-MB-231 Cells	CSCs	L929
CPT	36.9 ± 1.6	223.7 ± 13.3	15.9 ± 1.3
HA-capped AuNPs	34.8 ± 1.8	181.1 ± 15.6	935.97 ± 30.8
HA-capped AuNPs + L	22.4 ± 1.4	97.7 ± 5.6	495.1 ± 25.9

## Data Availability

The datasets used and/or analyzed during the current study are available from the corresponding author on reasonable request.

## Supplementary Materials

The following are available online at https://www.mdpi.com/article/10.3390/nano12193324/s1, Table S1. Primers information for PCR analysis; Figure S1. UV-vis spectra of HA capped AuNPs suspension (Au conc. = 75 µg/mL) at pre-determined sampling time and different storage temperature: 4 °C (A) or room temperature (B), reflecting the change in SPR peak. (C): The appearance of HA capped AuNPs suspension at different storage time. The left sample was kept at 4 °C and right one was at room temperature; Figure S2. The profiles of temperature variations of HA capped AuNPs suspensions upon exposure to NIR laser under different conditions (concentration of AuNPs, NIR laser treatment time and power density). The power density of NIR laser was fixed at 1 W/cm^2^ (A), 2 W/cm^2^ (B) and 3 W/cm^2^ (C), respectively; Figure S3. Change of MMP in MDA-MB-231 cells after treatment with HA capped AuNPs with/without NIR laser, reflected by JC-1 aggregates/monomers ratio. Scale bar = 50 μm; Figure S4. Expression of stemness-related genes such as *Nanog*, *Sox-2* in bulk MDA-MB-231 cells or CSCs, according to a RT-PCR assay (A). Relative mRNA level for each gene were all normalized by internal control *GAPDH* and shown in respective bar graphs (B). ** p* < 0.05, *** p* < 0.01 between bulk MDA-MB-231 cells and CSCs. Figure S5. Inhibitory effect of different drugs at various concentrations against MDA-MB-231 CSCs, according to a MTT assay, drug-cell interaction time was 24 h.

## Abbreviations

CSC, Cancer stem cells; AuNPs, Gold nanoparticles; HA, Hyaluronic acid; CPT, Camptothecin; NIR, Near infrared ray; SPR, Surface plasmon resonance; MMP, Mitochondrial membrane potential; CTAB, Cetyltrimethyl ammonium bromide; RT-PCR, Reverse transcription-polymerase chain reaction; MTT, 3-(4,5-Dimethylthiazol-2-yl)-2,5-diphenyltetrazolium bromide; DMEM, Dulbecco’s modified eagle medium; FBS, Fetal bovine serum; FTIR, Fourier transform infrared spectroscopy; ICP-OES, Inductively coupled plasma optical emission spectrometer; PDI, Polydispersity index; DLS, Dynamic light scattering; DI, Deionized; OD, Optical density; RIPA, Radioimmunoprecipitation assay buffer; SDS-PAGE, Sodium dodecyl sulfate polyacrylamide gel electrophoresis; ECL, Enhanced chemiluminescence.
