# Supplementary material for: Green Synthesis of Gold Nanoparticles and Study of Their Inhibitory Effect on Bulk Cancer Cells and Cancer Stem Cells in Breast Carcinoma
Source: Nanomaterials (Basel). 2022 Sep 24;12(19):3324. doi: 10.3390/nano12193324 (PMC9565927; doi:10.3390/nano12193324)
Supplement: Supplementary file 1 [file nanomaterials-12-03324-s001.zip › nanomaterials-1901058-supplementary.pdf]

nanomaterials

Supplementary Materials

Green Synthesis of Gold Nanoparticles and Study of Their Inhibitory Effect on Bulk Cancer Cells and Cancer Stem Cells in Breast Carcinoma

Jihui Wang [1,2], Na Liu [2], Qing Su [2], Yulong Lv [2], Chang Yang [2] and Honglei Zhan [2,*]

[1] School of Chemical Engineering and Energy Technology, Dongguan University of Technology, Dongguan 523808, China
[2] Department of Bioengineering, School of Bioengineering, Dalian Polytechnic University, Dalian 116034, China
* Correspondence: zhanhonglei121@163.com; Tel.: +0411-86324050

Table S1. Primers information for PCR analysis.

Primers.	Sequences (5' to 3')
GAPDH-F	ATGGCACCGTCAAGGCTGAG
GAPDH-R	GCAGTGATGGCATGGACTGT
Bax-F	ACCAAGAAGCTGAGCGAGTGT
Bax-R	ACAAACATGGTCACGGTCTGC
Bcl-2-F	GACTTCGCCGAGATGTCCAG
Bcl-2-R	CAGGTGCCGGTTCAGGTACT
P53-F	AAGGAAATTTGCGTGTGGAG
P53-R	TTCTGACGCACACCTATTGC
Caspase 3-F	CATACTCCACAGCACCTGGTTA
Caspase 3-R	ACTCAAATTCTGTTGCCACCTT
Nanog-F	GCAGGCAACTCACTTTATCC
Nanog-R	CCCACAAATCACAGGCATAG
Sox-2-F	CATCACCCACAGCAAATGAC
Sox-2-R	CAAAGCTCCTACCGTACCACT

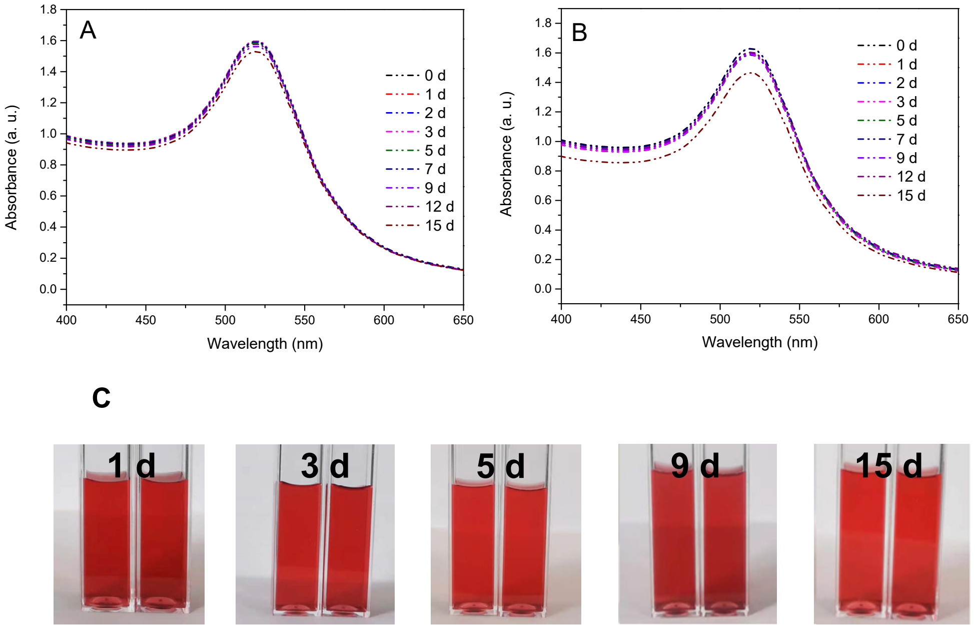

Figure S1. UV-vis spectra of HA capped AuNPs suspension (Au conc. = 75 μg/mL) at pre-determined sampling time and different storage temperature: 4 °C (**A**) or room temperature (**B**), reflecting the change in SPR peak. (**C**): The appearance of HA capped AuNPs suspension at different storage time. The left sample was kept at 4 °C and right one was at room temperature.

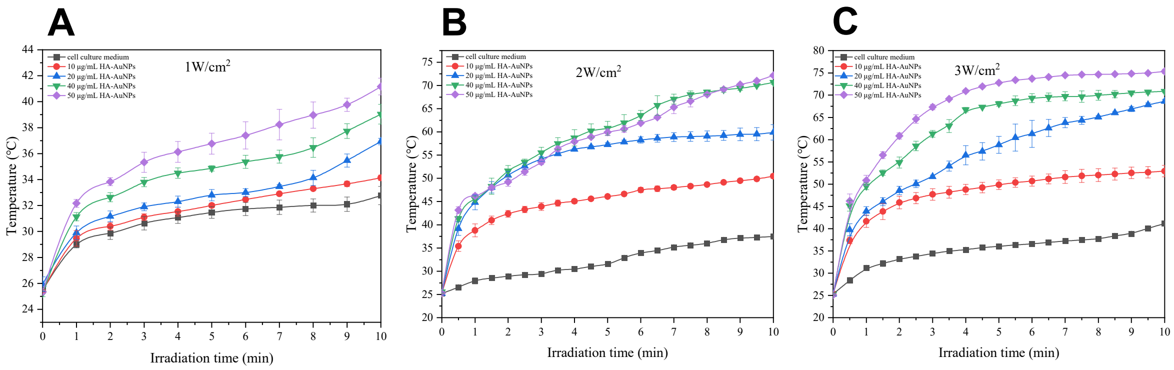

Figure S2. The profiles of temperature variations of HA capped AuNPs suspensions upon exposure to NIR laser under different conditions (concentration of AuNPs, NIR laser treatment time and power density). The power density of NIR laser was fixed at 1 W/cm² (**A**), 2 W/cm² (**B**) and 3 W/cm² (**C**), respectively.

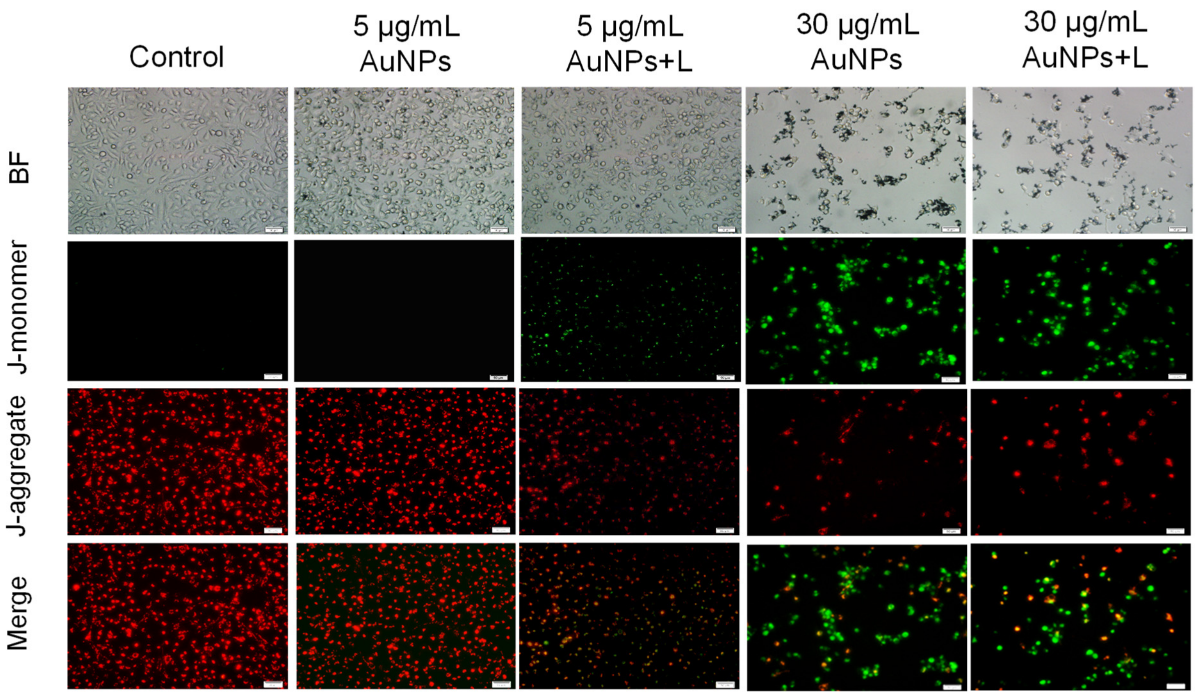

Figure S3. Change of MMP in MDA-MB-231 cells after treatment with HA capped AuNPs with/without NIR laser, reflected by JC-1 aggregates/monomers ratio. Scale bar = 50 μm.

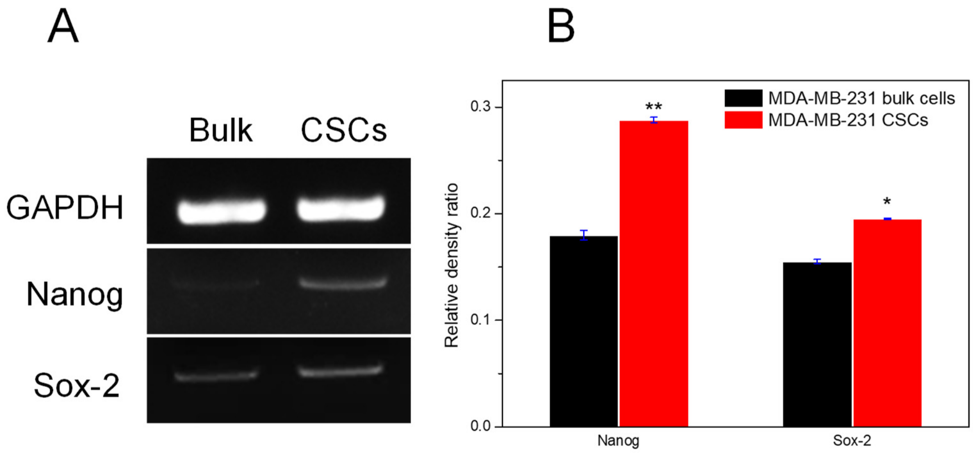

Figure S4. Expression of stemness-related genes such as *Nanog*, *Sox-2* in bulk MDA-MB-231 cells or CSCs, according to a RT-PCR assay (**A**). Relative mRNA level for each gene were all normalized by internal control *GAPDH* and shown in respective bar graphs (**B**). $^*p < 0.05$, $^{**}p < 0.01$ between bulk MDA-MB-231 cells and CSCs.

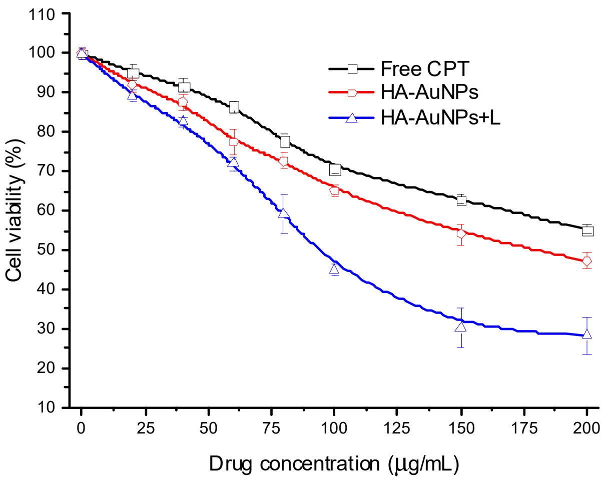

Figure S5. Inhibitory effect of different drugs at various concentrations against MDA-MB-231 CSCs, according to a MTT assay, drug-cell interaction time was 24 h.
